# Morphological changes in ICP pulse waveform as potential markers for early determination of external ventricular drain clamping trial outcome

**DOI:** 10.1186/2045-8118-12-S1-O3

**Published:** 2015-09-18

**Authors:** Jorge Arroyo Palacios, Maryna Rudz, Richard Fidler, Wade Smith, Nerissa Ko, Marvin Bergsneider, Xiao Hu

**Affiliations:** 1 University of California, San Francisco, USA; 2 University of California, Los Angeles, USA

## 

External ventricular drains (EVD) are widely used to measure and manage intracranial pressure (ICP) for aneurysmal subarachnoid hemorrhage (aSAH) patients. After several days of use a decision is made to remove the drain or replace it with an indwelling shunt. This involves a “clamping trial” whereby the EVD is clamped and transduced and pre and post clamping CT imaging is performed to observe any change in ventricular size and clinical status. This practice may lead to prolonged hospital stay, extra radiation exposure, and neurological insult. The present study aims to apply a widely validated morphological clustering analysis of ICP pulse (MOCAIP) algorithm to detect signatures from the pulse waveform to differentiate an intact CSF circulatory system from an abnormal one during EVD clamping. We hypothesize that an intact CSF system should be stable and pulses with a similar mean ICP level should have similar shapes.

This is a retrospective study with 43 out of 107 adult patients that satisfied the inclusion criteria of ICD9 code for SAH, admitted to UCSF Medical Center between 03/2013 and 08/2014, and exclusion criteria of absence of reported EVD clamping trial and ICP signal recordings shorter than 2 hours. By reviewing the clinical notes and pre/post brain imaging results, 25 patients were determined with an intact CSF circulatory system (group A) and 18 patients with an impaired one (group B). Blind to the chart review, the MOCAIP algorithm was applied to the ICP signal recordings to form a series of artifact-free dominant pulses. Then, for each pulse, other pulses with similar mean ICP were identified. Finally, the Euclidean and geodesic inter-pulse distances were calculated in a 4 hours moving window.

ANOVA analyses showed a significant difference on the standard deviations of the Euclidean (p<0.001, η2=0.29) and geodesic distance (p=0.001, η2=0.22), between groups A and B. Mann-Whitney U tests were used on the (non-normally distributed) mean distances, showing a significant difference on the Euclidean (p=0.007, r=0.41) and the geodesic distance (p=0.02, r=0.36) between the two groups of patients. The area under the ROC curve was 0.79 and 0.78 for the standard deviations of the Euclidean and geodesic distance, and 0.74 and 0.71 for the mean Euclidean and geodesic distance respectively.

Patients with an impaired CSF system showed not only a larger mean but also a larger variability of the inter-pulse distances, indicating frequent changes on the morphology of the pulses. The variability of morphological distances was the best predictor of the effect of EVD clamping trial. This technique may provide a method to rapidly determine if a patient will need surgical placement of an indwelling shunt or can simply have the EVD removed following SAH.

